# De-identified linkage of data across separate registers: a proposal for improved protection of personal information in registry-based clinical research

**DOI:** 10.1080/03009734.2018.1527420

**Published:** 2019-02-07

**Authors:** Tomas Snäckerström, Christian Johansen

**Affiliations:** Uppsala University, Uppsala Clinical Research Center, Uppsala, Sweden

**Keywords:** Data linkage, data protection, ethics, registry research

## Abstract

Over the last decades the advent of digital documentation has provided research communities with valuable resources of information for clinical research. To utilize the potential of information about patients, their health care, and its outcome that is already available in different registers, the possibility to cross-reference information from different registers is inevitably required. When performing linkage, we are currently forced to disclose information of participating subjects either to the administration of the other register(s) or to the researcher. Considering the increased concern of issues around personal integrity, this is a limitation that affects the ethical implications of proposed research and that might in the end affect the willingness of subjects to participate in registers. For this reason we propose a different methodology for performing cross-referencing, one that effectively prevents information leakage between the different organizations participating in linking the data. We believe that it is possible to use commonly adopted technologies within the area of data security and encryption to perform linkage without disclosing any sensitive information between different participants. In this paper we demonstrate how common techniques of encryption could be implemented to achieve that and furthermore significantly simplify discovery and feasibility surveying ahead of studies.

## Background

The widespread adoption of electronic documentation in health care has brought a huge potential to perform research by analyzing data from existing sources. Ranging from scientific cohorts, clinical quality registers, and health-care data to general data from personal devices, the potential for novel research is growing beyond what we could have imagined just a short time ago. In Sweden the population is furthermore comparatively well covered by registers with high quality and rich content, meaning that individual subjects are often better documented than elsewhere (1).

The documentation is, however, fragmented in separate registers of different types, with different purposes and in different legal domains. In order to fully study any specific population one needs to cross-reference data between the registers in question. The procedure of cross-referencing is, however, one that raises significant legal and ethical concerns (1 p. 15). The partitioning of information in separate organizations serves to mitigate the risk of gathering too much information about a person in one single place. Linking information between registers again is obviously not uncontroversial and needs to be done with caution.

With the adoption of the new General Data Protection Regulation (2) there is an increased awareness of integrity issues, and the continued availability of clinical data requires development of tools that safeguard the integrity of subjects while still allowing research to progress in the interest of the very same subjects. ‘Security by design’ is now a mandatory guideline on the design of IT systems that manages personal information of any kind—and in particular, sensitive data (3).

In clinical research it is common practice that data are used in a de-identified form, either anonymized or with pseudonyms replacing identifiers. This becomes problematic in the case of cross-referencing. In the current situation it is not possible to cross-reference without disclosing the identity of the subject either to the researcher or between the registers themselves, which really puts the ethical issues in focus. Within Cohorts.se we are therefore proposing the development of technical infrastructure that facilitates distributed analysis and use of safe cross-referencing that avoids leakage of sensitive data between participating organizations.

## The proposal

It should be mentioned that our idea is not primarily a novel technical solution. Nor does it require development of new software or use of cutting-edge technology. What we are proposing is rather about using well established technologies in a new area. We will not go into great detail regarding cryptography; it is arguably one of the most complex areas within the field of computer science and is well covered in the literature (4–6). In this paper we will only present some general principles and concepts that are essential to establish for the sake of the presentation.

The area of cryptography is centered around two generic problems in all forms of remote communication: secrets and trust—*secrets* as in the ability to ensure that only the intended recipient can read your message even though the message must be carried by untrusted messengers or might be eavesdropped on in the transport to the recipient; and *trust* in the sense that when received there has to be means for the recipient to verify the authenticity of the message, that it actually is from the sender, and that it has not been manipulated in any way. Both of these requirements are challenging but currently solvable ones.

In cryptography there are two main classes of methods: symmetrical and asymmetrical encryption algorithms. The symmetrical class of algorithms uses a key and some method of substituting characters based on the key. The asymmetrical algorithms use a pair of keys and non-reversible functions to create encrypted messages. One part of the key can decrypt messages if, and only if, they are encrypted with the matching part of the pair.

The symmetrical algorithms are significantly easier to compute but require that a key is agreed upon prior to sending the messages. In the case of asymmetrical algorithms the strict relationship between the key pairs can be used to address the question of authenticity of the messages. One part of the key is made public, and the other is kept secret. As messages encrypted by one part can only be decrypted by the other, the fact that one part is public becomes a means to assure whether it was the matching private key that encrypted the message in question. The two methods are usually applied in combination. Asymmetrical methods are used to communicate a key that is then used to symmetrically encrypt the messages.

We recognize that these attributes could be used to create a system where cross-referencing between different registers can be performed without the need to disclose personal information between any of the participants. We use the aspect of secrets when encrypting the identifiers, making them impossible to reverse, and we use trust in all of the remote communication for different systems to identify themselves to each other (‘authenticate’ in technical terminology).

Beyond the question of technology there is also a requirement to create an organizational structure for the administration and management of the required services in the proposed infrastructure. Our strategy relies on the ability to separate two required tasks into different administrations. Obviously, everything is dependent on the assumption that it can be regulated and that the administrations in question do not violate the rules. One should keep in mind that if we cannot rely on participating administrations to act according to rules there is no way to protect data in any system, existing or in the future.

Naming may need improvement, but for this discussion we call the services an ‘issuing service’ and a ‘key service’. The issuing service is responsible for overall orchestration of communication between the participating systems. The key service is only involved in creating and guarding the encryption keys during the processing. It is imperative that the key service only deals with the tasks around keys and that the issuing service is truly separated from the key service.

To explain the concepts involved we will present a walk-through of a hypothetical scenario. We want to stress that what we want to achieve is to minimize information leakage between different participating roles and organizations in this, yet providing the researcher with the ability to receive data that can be linked.

Researcher ‘Rachel’ has an idea about studying the correlation between two treatments or how one affects the outcome of the other. For this she needs to combine data from two different registries that both include data that are considered sensitive personal information. Register A may not disclose personal data to register B and vice versa. Rachel needs to receive data in an anonymized form and yet she needs to be able to link data between the two registers that relate to the same subject.

If neither register can send data to each other, nor send it to Rachel, it becomes intuitively attractive to designate a third party that could do the reference linkage. This has been suggested before but is really not helping the situation as it only creates a new entity that gets all the combined data instead. This is where the separation of key management as a distinct service, separate from handling of data, becomes a possible solution.

**Figure 1.**
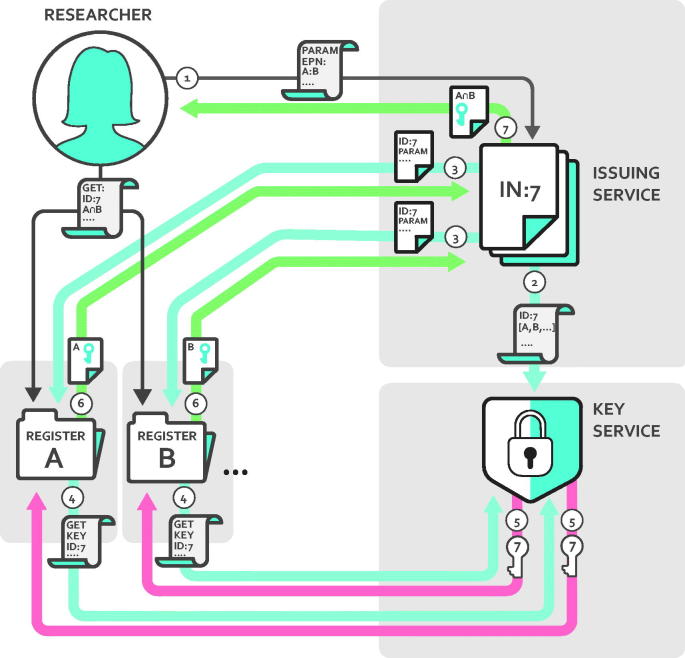
Illustration of the communication between participants involved when performing data linkage over different organizations. Any communication outside of what is illustrated is assumed to be prohibited and prevented by technical means.

In the following section there will be a description of the procedure of providing a dataset to Rachel without disclosing any common identifiers, as illustrated in Figure 1. In our proposed system Rachel creates a request for linkage to the issuing service in step one. We can now assume that there is a degree of administration required to verify ethical approvals as such, but once that is managed the issuing service creates an issue or a ‘job’ for the proposed data linkage and gives it an ID (‘IN:7’ for issue number 7 in Figure 1). In this job the requested registries are identified from what has been requested in the application (and given ethical approval).

In the second step the issuing service then requests that the key service creates a key for the job with the specific ID and tells the key service which registries are participating in the linkage job. The key service confirms to the issuing service that the key is created, but obviously keeps the key secret. The third step is where the issuing service sends independent requests to the registries in question, presenting information about the ID of the job, any parameters provided by Rachel (possible selection parameters, columns requested, etc.), and if required a reference to where to acquire the key.

Each registry individually requests the key for the job providing the reference ID they got from the issuing service, indicated as the fourth step. The key service authenticates the register systems by referencing the list of registers that the issuing service provided when requesting the job. If successfully authenticated, the common key is responded to the requesting register. At this point, the register is applying the encryption on all the relevant identifiers of their set of subjects, doing so independently and using the key and an encryption algorithm that was specified by the issuing service at the earlier step three.

Given that a common algorithm is used, all participating registers will then have created pseudonyms for their identifiers that are unique and similar for any given personal identifier that is common across registries, and that, without access to the key, cannot be reversed using any known computational method.

In step six the registries, independently of each other, report back their respective list of encrypted identifiers to the issuing service along with the reference for the job. When all the participating registries have reported back, the issuing service can easily distinguish the set of identifiers that are common for all participating registries (expressed as the intersection of A and B, A∩B). Note that the issuing service only gets the encrypted identifiers, but can still distinguish which identifiers are common in the sets it has received, as any given identifier will have the same encrypted representation. The seventh and last step is where the issuing service returns the set of encrypted identifiers that are common for the registries, the intersection, to Rachel along with a ticket identifying her as the rightful recipient of the extracted dataset.

With that, Rachel has a set of identifiers that are unique to her request and that can be used to request the actual data from each register. Doing so for all registers gives her tables from each participating registry which has common identifiers for the participating people but that still does not identify them directly. The only caveat remaining is the risk of indirect identification by the actual data, but that problem exists regardless of method for linkage and needs to be considered in the ethics review. It is reasonable to assume that the issuing service will need to be a point where a certain degree of control is performed, thus it is important that it is managed by a capable administration.

Summing up the rather elaborate description above we would like to point to the key aspects of the proposed solution. First, and most importantly, no information about the content in any of the registries ever leaves the registry itself. The encryption is done by the registry using reference implementations if standardized crypto-algorithms and only encrypted identifiers are communicated externally. There is an ongoing evolution of algorithms, but for this purpose there are alternatives that can be regarded as secure (7,8). Second, the registries can remain oblivious of the other registries involved. The registry solves its task without communication with any other registry involved. As a matter of fact, it does not need to know which or even how many registries take part in a job. Third, the key service never handles any data or receives any identifiers. It only distributes keys to securely authenticated clients. And finally, the system can also be created such that when the issuing service has received a set of encrypted identifiers from all participating registers in the job, it can order the key service and registers to destroy the key, prior to handing the list of encrypted identifiers to the researcher. This means that when the identifiers are sent to the researcher the key that was used in the intermittent process is already destroyed before data are given to the researcher. In this way, one can confidently ensure there is no foreseeable way of reversing the information in the identifier. This will also guarantee that data extracted in different research projects are not accidentally linked by the researcher after it has been handed out. Each ‘issue’ will have its unique and highly temporary key.

## Final thoughts

Although the proposed process might seem complicated when describing the theory, it should be said that all of what is happening is standard procedures and technologies used every day. Certificates are used to identify every server on the internet, and encryption is applied to ensure privacy whenever that is required, which is most of the times on the internet.

It should also be mentioned that all of the added communication can be automated and performed instantly. The technical overhead is minimal and can be performed in the background. For the user of the web it is not more complicated to access a service over a secure connection than over an unprotected one. Everything is set up in the background, and all that the user experiences is the green symbol that indicates that the server is authenticated as a trusted one by some authority the computer trusts.

Likewise, when Rachel presents her request for cross-referencing a set of registers there is obviously a need for an administrative process where her request and her ethical approval have been verified from a bureaucratic point of view. Once this has been granted, the rest of the process can be automated to the point where Rachel gets the links to where she can download her data. It requires an amount of development to create the systems, but as mentioned it is more about applying technologies and system components that are already available.

The automated aspect of the envisioned infrastructure could also enable some sought-after features. One of the most evident obstacles in designing a registry study is the difficulty in evaluating the feasibility of it. Often there is a need to know the approximate number of available subjects to assess the possibility of performing the study at all.

When cross-referencing is required it is obviously impossible to get an idea about the number of subjects that are common between a set of registers without doing the actual linkage. This means that in the current situation the researcher has to speculate on an idea, go through the process of acquiring ethical approval and the administrative process to get the linked dataset in order to discover whether or not there are enough subjects to perform a study. With an infrastructure such as envisioned above, one could quickly answer such questions without disclosing personal information to anyone.

The ability to answer such questions prior to initiating a process of ethical approval would not only give the researchers a tool to investigate the feasibility of their ideas, it would save the ethics committees the work required to decide upon research questions that are not even possible to investigate practically. It would even provide an important piece of information to the ethics processing itself. Showing that there is a large intersection between clinical areas is a fairly good indication that it covers an area that has a medical value to investigate.
